# The association between diet and sleep with internalising symptoms in young athletes: a serial multiple mediation models

**DOI:** 10.3389/fnut.2024.1382068

**Published:** 2024-08-27

**Authors:** Yun Gao, Lei Wang

**Affiliations:** ^1^Department of Physical Education, Shanghai Jiao Tong University, Shanghai, China; ^2^School of Physical Education, Shanghai University of Sport, Shanghai, China

**Keywords:** diet, sleep, internalising symptoms, athletes, mediation

## Abstract

**Introduction:**

Athletes frequently experience anxiety and depression at rates similar to or higher than non-athletes. A balanced diet can alleviate athletes internalizing symptoms. Moreover, diet and sleep are all associated with internalising symptoms. Our study investigates how sleep quality mediates the impact of dietary habits on internalizing symptoms in athletes.

**Methods:**

The current cross-sectional study involved 758 Chinese young athletes. The adapted Australian Athletes Diet Index was used to assess dietary patterns, and sleep was measured using the Athletes Sleep Screening Questionnaire. The Generalised Anxiety Disorder 7 scale and the Patient Health Questionnaire 9-item scale were used to assess symptoms of anxiety and depression. Structural Equation Modelling (SEM) analysis was used to examine the mediating role of sleep quality in young athletes. Raw scores of chronotypes, sleep quality, anxiety and depression were calculated for the statistical analysis.

**Results:**

No significant indirect effects were found in adolescents. In adult athletes, diet mediated the relationship between chronotype and sleep quality (*β* = −0.028, *p*  <  0.001). Sleep quality mediated the association between diet and anxiety (*β* = −0.060, *p* = 0.001), and depression (*β* = −0.076, *p* = 0.001). Additionally, diet and sleep quality mediated the association between chronotypes and anxiety (*β* = −0.028, *p* = 0.001), and depression (*β* = −0.028, *p* = 0.001).

**Conclusion:**

Sleep quality mediated the relationship between dietary patterns and internalising symptoms in young adult athletes. Additionally, diet and sleep quality mediated the association between chronotypes and internalising symptoms in young adult athletes.

## 1 Background

The main feature of internalising symptoms is the disturbance of mood or emotional state (1). These symptoms typically manifest as anxiety, depression, mood disorders, and social withdrawal (2, 3). These symptoms are characterised by negative emotional states and a lack of visible, externalised behaviours (4). In 2019, there were 280 million individuals worldwide experiencing depression and 301 million suffering from anxiety disorders (5). The prevalence of internalising symptoms is also notable in athletes. Recent studies suggest that elite athletes, due to the unique pressures of competitive sports, are particularly vulnerable to mental disorders (6, 7). A systematic review revealed that the prevalence of anxiety and depression in athletes varies but is often higher than or comparable to their non-athlete counterparts (6). Internalising symptoms can significantly impact an athlete’s physical performance and mental health. Depression and anxiety, for example, can lead to decreased motivation, impaired concentration, and suboptimal performance in sports (8). The high demands and stress associated with competitive sports can exacerbate these internalising symptoms, further affecting athletes’ overall well-being and career trajectory (9, 10).

To date, there is a limited number of studies focused on the relationship between diet and internalising symptoms in athletes. However, several studies indicated a positive association between better dietary habits and greater mental health. Dietary habits can influence mental health, where a balanced diet is associated with a reduced risk of depression and anxiety (11, 12). Previous studies suggested that a healthy diet rich in fruits, vegetables, and protein intake has benefits for reducing internalising symptoms in both the general population and athletes (12, 13). Conversely, poor dietary habits were associated with mental disorders in athletes (14). For instance, diets high in processed foods and sugars have been linked to increased risk of depression and anxiety (11, 12). Diet is one of the foundations for reaching athletic performance, with athletes’ dietary strategies, particularly those before and after competitions, being essential for recovery and adaptation (15). Effective dietary can enhance the adaptive response to fatigue, promote muscle function and boost exercise tolerance (16). Therefore, the monitoring of diets is important for athletes’ competition performance (17). However, scientific evidence indicates that there are some issues in the dietary behaviours of athletes. Specifically, there is an imbalanced intake of vitamins, including insufficient Vitamin D and excessive levels of phosphorus, iron and zinc (18). Elite athletes also demonstrate excessive consumption of vitamins, meat and sweets compared to non-elite athletes (19). Alcohol consumption among athletes poses a significant dietary problem (20). Research suggests that an athlete’s diet can impact their sleep, stress and overall well-being (21). Moreover, the understanding of nutrition among national-level athletes is profoundly limited and poorer than that of the general population of the same age (22, 23). Furthermore, a comparison between elite and non-elite athletes did not show substantial benefits in nutritional knowledge (24).

Sleep and internalising symptoms are closely related in athletes (25–28). Furthermore, sleep quality is even more closely linked to athletes’ mental health and is a significant predictor of internalising symptoms in athletes (29). Sleep deprivation is a contributing factor to impaired mental health in elite athletes (30). Chronic sleep deprivation and accumulated sleep debt can lead to severe mental wellness issues in athletes (31). The connection between sleep and mental health can be achieved by enhancing the recovery of the immune and nervous systems (32). Conversely, poor sleep quality and quantity have been consistently linked to higher levels of anxiety and depression among athletes (33). A longitudinal study on athletes indicated that those with poor sleep patterns were more likely to report symptoms of depression and anxiety, compared to those with adequate sleep (34). More importantly, diet plays a crucial role in the quality of sleep for athletes (35). It has been known that nutritional choices can either promote restorative sleep or contribute to sleep disturbances (35). Scientific evidence has also shown that a diet high in carbohydrates can facilitate the onset of sleep, while high-fat diets may adversely affect sleep quality (32, 36). Moreover, one qualitative review synthesised the effect of nutrition interventions on athletes’ sleep and indicated that sleep deprivation may lead to alterations in dietary habits (28).

The health behaviours of elite athletes are an ongoing concern, encompassing aspects such as diet and sleep. Athletes’ health behaviours can impact their training status and even directly affect their performance in competitions, ultimately influencing the trajectory of their careers (19). Therefore, the health behaviours and mental well-being of athletes during the Olympic preparation cycle are crucially important for optimising their performance (37). Although diet and sleep are associated with internalising symptoms, there is few studies have explored their interactive relationship in athletes. This gap is particularly notable given the high prevalence of mental health issues among athletes and the potential impact of these factors on their performance and well-being. The current study aims to address this gap by exploring how dietary habits and sleep patterns collectively influence internalising symptoms in athletes. Understanding the role of sleep as a mediator in this relationship is crucial, as it may offer insights into effective strategies for improving athletes’ mental health and, consequently, their sports performance. Therefore, the primary aim of this study is to investigate the association between diet and sleep with internalising symptoms in young athletes. Moreover, this study also explores the interactive relationship between unhealthy behaviours and internalising symptoms in young athletes.

## 2 Methods

### 2.1 Study design

This study was conducted using an online survey developed through the web-based questionnaire tool “Wen Juan Xing” (WJX.cn). The survey consisted of four sections: demographic data, health behaviours, and mental health. Participants were obtained by contacting coaches of university-level sports teams. Before participating in the study, athletes were provided with information about the research, and their informed consent was obtained. For athletes under the age of 18, parental or coach consent was required for participation in the survey. This study received approval from the Ethics Review Committee of the Shanghai University of Sport (Approval No. 102772022RT113).

Data were collected anonymously, and aggregate statistical analysis results were shared with the athletes or their guardians. The data collection was conducted from October to December 2022. Most of this period coincided with China’s COVID-19 epidemic prevention phase. On December 6, China announced new epidemic prevention regulations, initiating the comprehensive relaxation of epidemic control measures across society. However, the implementation of these measures continued in various locations until the end of December. Throughout the duration of this study, the training regimes of the athletes surveyed remained normal.

### 2.2 Participants

The sample size for this study was calculated using the G-power sample calculator, ensuring a probability level (*α* err prob) of *p* = 0.05, a small-to-medium effect size *d* = 0.3, and a statistical power (1-*β*) of 0.90, which necessitated a minimum sample size of 109. To ensure a sufficient number of athlete participants, there were no restrictions on the level or age of the athletes participating in the study, other than being between the ages of 13 and 29. The athletic levels considered were National First-Class, National Elite, and International Elite. Data from athletes below National First-Class or those not meeting the criteria (non-athletes) and duplicate entries were excluded.

According to the standards of the General Administration of Sport of China, National First-Class Athletes are those who meet the standards in national school leagues or national youth U-series competitions. National-Level Athletes are those qualified to participate in national competitions, while International-Level Athletes generally refer to those qualified to participate in international or intercontinental competitions. The standards for athlete levels vary by sport, as detailed in the standards of the General Administration of Sport of China (38).

### 2.3 Measures

This study targeted high-level competitive athletes in China, specifically those who have been engaged in long-term sports training and have participated in provincial-level or higher competitions. The survey incorporated internationally recognised scales suitable for high-level athletes, facilitating the identification of health behaviour issues in this group. The comprehensive questionnaire included the following sections.

#### 2.3.1 Diet measurement

The dietary behaviour questionnaire was adapted from the core nutritional section of the Australian Athlete Diet Index (ADI). The ADI is a scale specifically designed for assessing the nutritional behaviours of athletes (39). It demonstrates good reliability (ICC = 0.80, 95% CI 0.69, 0.87; *p* < 0.001), and Bland–Altman plots (mean 1.9; limits of agreement −17.8, 21.7) did not show systematic bias (*y* = 4.57–0.03 × *x*) (95% CI −0.2, 0.1; *p* = 0.70) (40). Due to regional and cultural differences, the structure of the ADI core nutrition section was used, with item content referencing the “Chinese Elite Athlete Nutrition Guide” for the dietary requirements of athletes. The athletes were required to recall their dietary patterns over the past week. The questionnaire contains 13 items, covering the total variety of fruit, vegetables, grains, whole grains, dairy products, low-fat dairy products, total meat, poultry, fish, sweets, processed meats, takeout and dining out, and alcohol intake. The adapted questionnaire was retested for reliability in the athlete population, showing a test–retest reliability (ICC = 0.711, 95% CI 0.69, 0.87; *p* < 0.001). Higher raw scores represent healthier diet behaviours.

#### 2.3.2 Sleep measurement

The Athlete Sleep Screening Questionnaire (ASSQ) was used to assess sleep, including sleep quantity, quality, timing, insomnia, sleep-disordered breathing, and travel-related sleep issues, categorising athletes into four groups based on a “Sleep Difficulty Score”: none, mild, moderate, and severe clinical problem. The ASSQ shows good agreement with personal assessments by physicians specialising in elite athlete sleep (Cohen’s kappa = 0.84), with a diagnostic sensitivity of 81%, specificity of 93%, positive predictive value of 87%, and negative predictive value of 90%. It is the only universally used sleep screening questionnaire for athletes and has been clinically validated (41, 42). The ASSQ is a 16-item multiple-choice questionnaire that calculates the “Sleep Difficulty Score (SDS)” using five questions based on total sleep time, sleep satisfaction, and symptoms of insomnia. The SDS, along with scores from other questions assessing circadian rhythm and sleep-disordered breathing, are translated into specific intervention recommendations. The ASSQ has been proven effective and reliable in the athletic population (41). The first five items of the ASSQ assess sleep disorders (e.g., In the past month, how many hours do you sleep at night?), with a scoring system of 4 and 5 points. A total score of 1–17 is calculated, with ≥8 indicating the presence of a sleep disorder. Chronotype can be defined as an individual’s inherent propensity towards a preference for morningness or eveningness (43). In terms of ASSQ Chronotype, the raw score ≤ 4 indicates the athlete is an evening type.

#### 2.3.3 Anxiety and depression measurement

The Generalised Anxiety Disorder 7 (GAD-7) scale and the Patient Health Questionnaire 9-item (PHQ-9) scale were used to assess symptoms of anxiety and depression in athletes. The GAD-7 assesses anxiety through seven items, such as “Over the last 2 weeks, how often have you been bothered by the following problem? (1) Feeling nervous, anxious, or on edge. (2) Not being able to stop or control worrying” Each item is scored from 0 (“Not at all”) to 3 (“Nearly every day”), with a maximum score of 21. A total score between 0 and 21, with ≥10 indicating anxiety symptoms. The PHQ-9 measures depression through nine items, such as: “Over the past 2 weeks, how often have you been bothered by any of the following problems?” (1) Little interest or pleasure in doing things. (2) Feeling down, depressed, or hopeless. Each item is scored from 0 (“Not at all”) to 3 (“Nearly every day”), with a maximum score of 27. A total score of 0–27 is calculated, with ≥10 indicating depressive symptoms.

### 2.4 Data analysis

Descriptive statistics and correlations were calculated using IBM SPSS v26. The correlation analysis was controlled for variables such as age, sex, BMI, athlete grade, training hours/day, training days/week, and perceived training intensity. Structural Equation Modelling (SEM) analysis was conducted in IBM AMOS v24. The SEM analysis aimed to examine the relationships between diet, sleep quality scores, chronotype scores, and the variables of depression and anxiety. Dietary patterns were the independent variable. Sleep quality was treated as a mediating variable, with anxiety and depression as dependent variables. Since the given model met these criteria, maximum likelihood was used to calculate estimates. Following Kline’s recommendations, the chi-square test, Comparative Fit Index (CFI > 0.9), Goodness of fit (GFI > 0.9), Index Root Mean Square Error of Approximation (RMSEA < 0.05), and Standardised Root Mean Square Residual (SRMR < 0.05) were calculated to assess the goodness of fit for each model (44). Indirect effects were tested using a bias-corrected bootstrap with 5,000 bootstrap samples. Confidence intervals were calculated at the 95% level. The significance level was set at 0.05.

## 3 Results

The descriptive statistics of demographic, health behaviour, and mental health variables from the questionnaire, as well as the mean comparisons among subgroups, are presented in Table 1. In total, 815 online questionnaire responses were confirmed. After applying eligibility criteria, 758 of these were selected as the sample data for the study. Athletes from 22 different sports participated in the survey. The top five most represented sports were: Track and Field (22.0%), Diving (15.2%), Swimming (12.0%), Gymnastics (9.8%), and Tennis (8.6%). Finally, this study ultimately included questionnaire data from 758 athletes (19.36 ± 3.17 years). Of these, 388 were male (51.19%) and 370 were female (48.81%); there were 50 International Elite athletes, 274 National Elite athletes, and 434 National First-Class athletes. In the comparison between male and female samples, female athletes scored lower than male athletes in overall diet scores (52.69 ± 14.92 vs. 57.69 ± 13.61, *p* < 0.01), had higher scores in sleep difficulties (6.08 ± 2.92 vs. 5.59 ± 2.85, *p* < 0.05), and scored lower in late sleeping (reverse scoring: 6.37 ± 1.90 vs. 6.71 ± 1.86, *p* < 0.05). Female athletes also had higher scores in anxiety and depression than male athletes (5.51 ± 4.56 vs. 4.22 ± 4.22, *p* < 0.01; 6.45 ± 5.00 vs. 4.98 ± 4.73, *p* < 0.01), with the only non-significant statistical differences being in Training hours/day (*p* = 0.317) and Training days/week (*p* = 0.496). In the comparison among different athletic levels, significant statistical differences were observed in the age (23.06 ± 3.62 vs. 20.01 ± 3.25 vs. 18.52 ± 2.62, *p* < 0.001), diet (56.83 ± 13.14 vs. 53.11 ± 14.99 vs. 56.42 ± 14.15, *p* = 0.009), and anxiety (5.28 ± 5.51 vs. 5.39 ± 4.50 vs. 4.47 ± 4.23, *p* = 0.021) scores of International Elite athletes, National Elite athletes, and National First-Class athletes, while other indicators showed no significant statistical differences (*p* > 0.05).

**Table 1:** Descriptive statistics for demographic variables and the health questionnaires.

Variables	Total (*N* = 758)	Male (*N* = 388)	Female (*N* = 370)	*T* value	*p*	International elite (*N* = 50)	National elite (*N* = 274)	National first-class athletes (*N* = 434)	*F* value	*p*
Age	19.36 ± 3.17	19.76 ± 3.25	18.95 ± 3.04	3.540	<0.001	23.06 ± 3.62	20.01 ± 3.25	18.52 ± 2.62	64.335	<0.001
BMI	22.48 ± 8.15	23.54 ± 9.48	21.38 ± 6.31	3.667	<0.001	23.15 ± 4.88	22.14 ± 4.19	22.63 ± 10.11	0.482	0.618
Training hours/day	5.21 ± 10.87	4.82 ± 2.78	5.61 ± 15.29	−1.001	0.317	5.66 ± 1.79	5.18 ± 2.99	5.17 ± 14.16	0.046	0.955
Training days/week	5.82 ± 2.62	5.89 ± 2.31	5.76 ± 3.0	0.682	0.496	6.14 ± 1.29	5.99 ± 3.09	5.68 ± 2.40	1.635	0.196
Diet	55.25 ± 14.47	57.69 ± 13.61	52.69 ± 14.92	4.825	<0.001	56.83 ± 13.14	53.11 ± 14.99	56.42 ± 14.15	4.747	0.009
Chronotype	6.55 ± 1.88	6.71 ± 1.86	6.37 ± 1.90	2.481	0.013	6.08 ± 2.23	6.58 ± 1.76	6.58 ± 1.92	1.643	0.194
Sleep quality	5.83 ± 2.89	5.59 ± 2.85	6.08 ± 2.92	−2.341	0.019	5.84 ± 3.19	6.01 ± 2.94	5.72 ± 2.83	0.847	0.429
Anxiety	4.85 ± 4.44	4.22 ± 4.22	5.51 ± 4.56	−4.041	<0.001	5.28 ± 5.51	5.39 ± 4.50	4.47 ± 4.23	3.883	0.021
Depression	5.69 ± 4.91	4.98 ± 4.73	6.45 ± 5.00	−4.160	<0.001	5.60 ± 5.86	6.22 ± 4.98	5.37 ± 4.73	2.509	0.082

**p < 0.01, *p < 0.05. ASSQ, The Athlete Sleep Screening Questionnaire; SDS, Sleep Difficulty Score; Chronotype, Eveningness–tendency to sleep late; SMHRT-1, Sport Mental Health Assessment Tool 1.

The correlation coefficients among indicators are shown in Table 2. After controlling for variables such as age, sex, BMI, athlete grade, training hours/day, training days/week, and perceived training intensity, the athletes’ diet raw scores were significantly negatively correlated with sleep quality raw scores (*r* = −0.128, *p* < 0.01), and significantly positively correlated with chronotypes raw scores (*r* = 0.138, *p* < 0.01), anxiety raw scores (*r* = 0.091, *p* < 0.05), and depression raw scores (*r* = 0.099, *p* < 0.01). Sleep quality raw scores were significantly positively correlated with anxiety (*r* = 0.384, *p* < 0.01) and depression raw scores (*r* = 0.450, *p* < 0.01). There was also a significant positive correlation between anxiety and depression raw scores (*r* = 0.821, *p* < 0.01).

**Table 2:** Zero-order correlation of variables (*N* = 750).

Measure	Diet	SDS	Chronotype	Anxiety
SDS	−0.136**			
Chronotype	0.134**	−0.058		
Anxiety	−0.091*	0.386**	0.004	
Depression	−0.101**	0.451**	−0.034	0.821**

*Significant differences between groups, *p* < 0.05; **significant differences between groups, *p* < 0.01.

Tables 3, 4 display the direct effects of chronotypes, diet and sleep on anxiety and depression raw scores in young athletes. In adolescents, sleep quality was associated with anxiety (*β* = 0.431, 95%CI = 0.299–0.552, *p* < 0.01) and depression (*β* = 0.382, 95%CI = 0.289–0.563). In adults, chronotype was positively associated with diet (*β* = 0.167, 95%CI = 0.074–0.253, *p* < 0.01). Diet was negatively related to sleep quality (*β* = −0.168, 95%CI = −0.242 to −0.091, *p* < 0.01). Additionally, sleep quality was positively associated with anxiety (*β* = 0.359, 95%CI = 0.275–0.432, *p* < 0.01) and depression (*β* = 0.451, 95%CI = 0.377–0.522, *p* < 0.01).

**Table 3:** Direct effects of chronotype, diet and sleep duration on anxiety.

Path	Overall sample			Adolescents			Adults		
	Standardised estimates	95%CI		Standardised estimates	95%CI		Standardised estimates	95%CI	
		Lower	Upper		Lower	Upper		Lower	Upper
Chronotype → diet	0.152***	0.075	0.227	0.078	−0.077	0.235	0.167**	0.074	0.253
Chronotype → sleep quality	−0.058	−0.132	0.020	−0.066	−0.211	0.079	−0.047	−0.129	0.046
Diet → sleep quality	−0.135***	−0.199	−0.066	−0.053	−0.177	0.073	−0.168**	−0.242	−0.091
Diet → anxiety	−0.064	−0.132	0.001	−0.058	−0.166	0.055	−0.072	−0.144	0.011
Sleep quality → anxiety	0.382***	0.313	0.449	0.431**	0.299	0.552	0.359**	0.275	0.432

***p < 0.001, **p < 0.010, *p < 0.050.

**Table 4:** Direct effects of chronotype, diet and sleep duration on depression.

Path	Overall sample			Adolescents			Adults		
	Standardised estimates	95%CI		Standardised estimates	95%CI		Standardised estimates	95%CI	
		Lower	Upper		Lower	Upper		Lower	Upper
Chronotype → diet	0.152***	0.075	0.227	0.078	−0.077	0.235	0.167**	0.074	0.253
Chronotype → sleep quality	−0.058	−0.132	0.020	−0.066	−0.210	0.082	−0.047	−0.129	0.046
Diet → sleep quality	−0.135***	−0.199	−0.066	−0.053	−0.171	0.078	−0.168**	−0.242	−0.091
Diet → anxiety	−0.061	−0.122	0.001	−0.083	−0.197	0.032	−0.056	−0.129	0.021
Sleep quality → anxiety	0.445***	0.376	0.510	0.382***	0.289	0.563**	0.451**	0.377	0.522

***p < 0.001, **p < 0.010, *p < 0.050.

Tables 5, 6 show the indirect effects of chronotype, diet, and sleep quality on anxiety and depression raw scores. In adolescents, we did not find any significant indirect effects. In adults, diet mediated the relationship between chronotype and sleep quality (*β* = −0.028, *p* < 0.001). Sleep quality mediated the association between diet and anxiety (*β* = −0.060, *p* = 0.001), and depression (*β* = −0.076, *p* = 0.001). Additionally, diet and sleep quality mediated the association between chronotypes and anxiety (*β* = −0.028, *p* = 0.001), and depression (*β* = −0.028, *p* = 0.001).

**Table 5:** Indirect effects on anxiety.

Paths	Overall		Adolescents		Adults	
	Standardised estimates	*p*	Standardised estimates	*p*	Standardised estimates	*p*
Chronotype → diet → sleep quality	−0.020***	0.001	−0.004	0.296	−0.028***	0.001
Chronotype → diet → anxiety	−0.010*	0.039	−0.005	0.240	−0.012	0.067
Chronotype → sleep quality → anxiety	−0.022	0.134	−0.028	0.369	−0.017	0.314
Diet → sleep quality → anxiety	−0.051***	0.001	−0.023	0.395	−0.060***	0.001
Chronotype → diet → sleep quality → anxiety	−0.020***	0.001	−0.004	0.288	−0.028***	0.001

***p < 0.001, **p < 0.010, *p < 0.050. Model fit index (adults): chi-square (*χ*^2^/DF = 1.066, *p* = 0.302), SRMR = 0.013, RMSEA = 0.011, CFI = 0.999, GFI = 0.999.

**Table 6:** Indirect effects on depression.

Paths	Overall	Adolescents			Adults	
	Standardised estimates	*p*	Standardised estimates	*p*	Standardised estimates	*p*
Chronotype → diet → sleep quality	−0.020***	0.001	−0.004	0.296	−0.028***	0.001
Chronotype → diet → depression	−0.009*	0.036	−0.006	0.233	−0.009	0.124
Chronotype → sleep quality → depression	−0.026	0.142	−0.029	0.363	−0.021	0.317
Diet → sleep quality → depression	−0.060***	0.001	−0.023	0.373	−0.076***	0.001
Chronotype → diet → sleep quality → depression	−0.020***	0.000	−0.004	0.280	−0.028***	0.001

***p < 0.001, **p < 0.010, *p < 0.050. Model fit index (adults): chi-square (*χ*^2^/DF = 0.002, *p* = 0.964), SRMR = 0.001, RMSEA = 0.000, CFI = 1.000, GFI = 1.000.

## 4 Discussion

To the best of the author’s knowledge, this is the first study to explore the mediating role of sleep in the relationship between diet and internalising symptoms in young athletes. The results indicate that there are significant associations between chronotype and diet, diet and sleep quality, sleep quality and anxiety/depression. Furthermore, our findings revealed two significant mediating pathways: (1) diet → sleep quality → anxiety/depression and (2) chronotype → diet → sleep quality → anxiety/depression in young adult athletes. Additionally, we did not find any indirect effects in adolescent athletes.

Our findings suggest a positive association between chronotype and diet raw scores in young adult athletes, which is consistent with the previous studies (45–47). The previous study investigated adults aged 18–65 years and found that those with a morning chronotype were intended to consume higher energy, protein and fat intake, and reduced carbohydrate intake (45). Conversely, evening chronotypes consumed lower protein intake (45). In university athletes, a cross-sectional study indicated that evening chronotype athletes consumed higher amounts of carbohydrates, confectionary, and sweet beverages compared to their morning chronotype counterparts (46). Furthermore, Moss and colleagues found higher chronotype raw scores were linked to increased gain consumption (47). Morning chronotypes were identified as individuals consuming more breakfasts, while evening chronotypes displayed preferences for more snacks, caffeine, alcohol, and later eating patterns (45, 48, 49). A scoping review also suggested that evening chronotypes were associated with higher consumption of sweets, caffeine, and alcohol, as well as unhealthier behaviours such as delayed meal times, irregular breakfast eating, breakfast skipping and excessive calorie intake during the night (50).

The current study found that dietary patterns were associated with sleep quality in young athletes. This finding aligns with previous studies (47, 51). For instance, Moss and colleagues found endurance athletes who consumed fewer grains had poorer sleep quality (47). Cando and colleagues found nutrition intake was associated with both sleep duration and quality in elite female athletes (51). Additionally, our findings suggest a positive association between sleep quality and anxiety/depression raw scores in young athletes. This finding is consistent with several previous studies (52–54). Grandner and colleagues found that poor sleep quality was associated with a higher risk of anxiety (Unstandardised *B* = 0.459, 95%CI = 0.294–0.624, *p* < 0.0001) and depression (Unstandardised *B* = 0.52, 95%CI = 0.239–0.801, *p* = 0.0003) (53). Moreover, Potter and colleagues investigated 162 high school athletes and found poor sleep quality was associated with higher anxiety (*β* = 0.391, 95%CI = 0.263–0.520, *p* < 0.001) and depressive symptoms (*β* = 0.456, 95%CI = 0.346–0.565, *p* < 0.001) (54). We also found that sleep quality was associated with anxiety/depression in adolescent athletes. Adolescents may experience poor sleep quality due to maturational development (e.g., a circadian phase delay) and societal and psychosocial factors (e.g., increased bedtime autonomy, and screen time) (55, 56). It is well-documented that poor sleep quality is associated with higher levels of anxiety and depressive symptoms (54, 57). This can partially explain the association between sleep quality and anxiety/depression in adolescent athletes. Our findings also support the strongest association between sleep quality and internalising symptoms in young athletes. However, mediating analysis suggests that no direct effects are observed between diet and anxiety/depression, as well as chronotypes and sleep quality in young athletes.

In terms of indirect effects, the current study found that sleep quality mediated the relationship between diet and internalising symptoms in young adult athletes. The previous study has indicated that unhealthy dietary patterns in conjunction with inadequate sleep may constitute significant risk factors for the onset of mental health problems (58). Our study extends this finding by demonstrating that sleep quality mediates the association between dietary patterns and anxiety/depression in young adult athletes. Accordingly, diet can impact sleep through various mechanisms such as circulating gut hormones, stimulating the production of serotonin and melatonin, acting on GABAergic or serotonergic neurons, or other unidentified mechanisms such as the bioactive peptides and the nonprotein nitrogen fraction of diet (59, 60). For these reasons, unhealthier dietary patterns may lead to poor sleep quality in young athletes (61). Moreover, the relationship between sleep and anxiety may be explained by shared neurocircuity and there is a complex network of receptor systems, including dopamine, serotonin and adenosine (62). Additionally, sleep may impact depression via increasing inflammatory cytokines, biochemical pathways such as changes in cholinergic and monoaminergic neurons, genetic factors and circadian rhythm (63). Taken together, the relationship between diet and internalising symptoms may be explained by the effects of diet on sleep quality, which may, in turn, impact internalising symptoms in young athletes. Moreover, diet and sleep quality mediated the association between chronotype and internalising symptoms. According to existing evidence, morning chronotype people were more likely to have a healthier and regular lifestyle when compared to their evening counterparts (48, 49, 59). In comparison with the mediating effects of sleep quality, the indirect effect of chronotype on internalising symptoms is smaller than the mediating effect of sleep quality. This finding indicates that chronotype may be associated with insufficient sleep duration through poor dietary behaviours, which could subsequently be related to anxiety and depression. It implies that coaches, athletes, and other stakeholders should be aware that both a healthier diet and better sleep quality potentially have benefits for reducing the risk of internalising symptoms in athletes.

This study represents one of the initial attempts to examine the mediating effects of sleep quality in the relationship between diet and internalising symptoms in young athletes. However, several limitations should be considered when interpreting the findings. Firstly, a cross-sectional study design was employed to examine the relationship between diet, sleep and internalising symptoms in young athletes. More experimental studies are needed to establish the causality of this association. Secondly, self-reported measures were used to assess diet, sleep and internalising symptoms. To enhance our understanding of this association, further research would benefit from incorporating more objective measures. Thirdly, as the sample comprises Chinese young athletes, improving the generalisability of the results is recommended by including a larger sample size and participants from diverse cultural backgrounds. Lastly, given that the data collection was conducted between October and December 2022 during the period of COVID-19. The findings of the current study should be interpreted with caution.

## 5 Conclusion

The results suggest significant direct effects of chronotype on diet, diet on sleep quality, and sleep quality on anxiety/depression in young adult athletes. Moreover, the mediating models indicate that sleep quality mediated the relationship between dietary patterns and internalising symptoms in young adult athletes. Additionally, diet and sleep quality mediated the association between chronotype internalising symptoms in young adult athletes.

## Data Availability

The datasets presented in this article are not readily available because of the restrictions of ethics. Requests to access the datasets should be directed to Lei Wang: wl2119@163.com.
